# The potential role of community pharmacy staff in reducing patient delay in consulting with symptoms of rheumatoid arthritis: a qualitative study

**DOI:** 10.1186/s41927-022-00280-0

**Published:** 2022-08-24

**Authors:** Gwenda Simons, Nour Ismail, Karanbir Sandhu, Christian D. Mallen, Rebecca J. Stack, Sarah Pontefract, Karim Raza, Marie Falahee

**Affiliations:** 1 Rheumatology Research Group, Institute of Inflammation and Ageing (IIA), College of Medical and Dental Sciences, University of Birmingham, Birmingham, UK; 2 School of Pharmacy, College of Medical and Dental Science, University of Birmingham, Birmingham, UK; 3 Medical School, College of Medical and Dental Sciences, University of Birmingham, Birmingham, UK; 4 School of Medicine, Keele University, Keele, UK; 5 Institute of Clinical Science, University of Birmingham, Birmingham, UK; 6 School of Pharmacy, College of Medical and Dental Science, Institute of Clinical Sciences, University of Birmingham, Birmingham, UK; 7 Sandwell and West Birmingham NHS Trust, Birmingham, UK

**Keywords:** Pharmacists, Community pharmacy, Rheumatoid arthritis, Early treatment, Help seeking, Treatment delay

## Abstract

**Background:**

Rheumatoid arthritis (RA) is a chronic inflammatory arthritis which can cause joint damage and reduced quality of life. Early treatment of RA within 3 months of symptom onset is associated with improved clinical outcomes. However, this window of opportunity is often missed. One important contributing factor is patients with symptoms of RA delaying consulting their general practitioner (GP). Previous research indicates that patients with inflammatory arthritis are likely to visit pharmacies for advice before consulting their GP. Therefore, pharmacists are well positioned to identify patients with symptoms of early inflammatory arthritis and signpost them appropriately. This research examines community pharmacy staff’s knowledge, perceptions, and approaches to management of patients presenting with symptoms of RA in order to identify training needs and other opportunities for intervention to enhance the role of pharmacy staff in the pathway to care.

**Methods:**

Semi-structured interviews were conducted with 19 community pharmacy staff in the West Midlands (UK), during a 12-month period (2017–2018). The interviews were audio-recorded, transcribed, and analyzed using thematic analysis facilitated by NVivo 12**.**

**Results:**

There was considerable variation in knowledge and perceptions of RA and the need for early treatment amongst pharmacists and other pharmacy staff. The potential role of pharmacists and other pharmacy staff in reducing delay in help-seeking was also discussed. Four themes emerged from thematic analysis: (1) Variations in perceptions and knowledge about RA. (2) The role of the pharmacy in increasing public awareness about RA. (3) The role of the pharmacy staff in facilitating access to the GP. (4) Practical considerations for pharmacy-based interventions.

**Conclusion:**

Variability in knowledge and perceptions of RA amongst pharmacists, and amongst other pharmacy staff will affect effective signposting of suspected RA cases. This study identifies opportunities for enhanced training of community pharmacists and other pharmacy staff in relation to inflammatory arthritis as well as other pharmacy-based interventions, such as public awareness campaigns about RA and other musculoskeletal conditions. Together with existing referral services and other pharmacy-based initiatives this could result in enhanced signposting to GP consultation or other appropriate NHS services for inflammatory symptoms and reduced treatment delay.

## Background

Rheumatoid arthritis (RA) is a common chronic inflammatory disease with a prevalence in the UK of approximately 0.5–1% [1]. RA is characterized by synovial inflammation which can lead to long-term joint damage. RA can have a considerable negative impact on the quality of life of patients, is associated with reduced life expectancy [2] and presents a significant socioeconomic burden due to increased healthcare use and productivity loss [3].

According to National Institute for Health and Care Excellence (NICE) guidance and the European Alliance of Associations for Rheumatology (EULAR) recommendations, early treatment with disease modifying anti-rheumatic drugs (DMARDs) is recommended [4, 5]. Early treatment improves outcomes for people with RA, facilitating the achievement of clinical remission, reducing the rate of joint damage and improving the patient’s quality of life [2, 6, 7]. There is a ‘therapeutic window of opportunity’, during which the initiation of treatment is most beneficial in reducing joint damage and preventing future disability; this has been estimated to last between three and four months after the onset of symptoms [2, 4, 7–9]. However, only a minority of patients with RA are diagnosed and receive appropriate treatment within the recommended window. One UK study showed patients waiting 3 months (*Mdn* = 12 weeks; *IQR* 4–28 weeks) before seeking help for their RA symptoms from their GP [10], with a large proportion of patients thus missing the window of opportunity before being assessed by a rheumatologist. Indeed, although there is variation across countries, one pan-European study showed that the median time between symptom onset and assessment by a rheumatologist was 24 weeks instead of the recommended 12 weeks [11]. A detailed analysis of treatment delay in early RA patients in Belgium [12] showed similar delays (median time was 23 weeks, only 22% of patients were assessed by a rheumatologist within 12 weeks).

It has been demonstrated that an important factor contributing to delayed DMARD initiation is the delay between symptom onset and help-seeking by the patients themselves [10, 13–15]. Multiple factors underlie this patient delay. Whilst accurate recognition that symptoms are suggestive of a serious underlying condition is an important driver for early help seeking [16], many patients do not perceive their initial inflammatory joint symptoms to be serious or worthy of urgent medical attention [17]. Pain is a commonly reported symptom of early RA and patients often seek to self-manage this pain with over the counter (OTC) medications [18].

In a survey of the general public conducted in the UK, 40% of 1088 respondents reported that they would visit a pharmacy for advice before or instead of visiting a GP following the onset of RA symptoms (Unpublished data from the RAPID study [17]). This suggests that pharmacy staff and in particular pharmacists may be well positioned to signpost patients with suspected RA symptoms towards GP consultation. Pharmacists are widely viewed as a reliable source of information for health concerns as well as providing guidance about when GP consultation is appropriate [19]. In the UK, people are further actively encouraged and signposted to go to their pharmacy for minor ailments such as ‘aches and pains’ [20]. Systematic reviews of the impact of pharmacy-delivered public health interventions highlight positive effects on health outcomes [21, 22] and suggest that integration of pharmacists into primary care teams reduces GP appointments and leads to savings in overall health system and medication costs [23, 24].

There is evidence for the effectiveness of pharmacy-led interventions for musculoskeletal problems [25–27]. For example, a cluster randomized controlled trial in community pharmacies showed that pharmacists were able to initiate a multidisciplinary intervention to identify people with knee osteoarthritis, improve the utilisation of treatments, and improve patients’ function, pain, and quality of life [25, 26]. There is also evidence of the benefits of pharmacist-led intervention on cardiovascular risk in patients with chronic inflammatory disease [27].

In order for pharmacists to have a positive impact on the early diagnosis and initiation of treatment RA, it is vital that they have the appropriate knowledge to identify inflammatory symptoms, distinguish them from self-limiting musculoskeletal (MSK) conditions for which self-management would be appropriate, and signpost patients with the former towards rapid GP consultation. Many universities now provide training about the (early) symptoms of RA, in addition to the extensive training about RA medication. However, historically training in musculoskeletal disease across disciplines was not as comprehensive as it is now and so pharmacists who completed their training many years ago may have gaps in their knowledge around RA unless these have been filled by post qualification continuing professional development (CPD).

It is further important to assess perceptions and knowledge of RA in other members of the pharmacy team as well as their perceived role in signposting individuals with MSK symptoms as they will often have the initial contact with the patient and are, for example, asked about OTC medication, before being referred on to the pharmacist.

This qualitative interview study explores knowledge and perceptions of RA amongst pharmacists and other community pharmacy staff, and their views about their role in the identification and management of patients presenting with symptoms of RA and other MSK conditions. The main objective was to identify specific training needs and other opportunities for pharmacy staff to have an enhanced role in reducing treatment delay.

## Methods

### Study design

This was a qualitative interview study which used an inductive approach [28]. The multidisciplinary research team (including pharmacists, rheumatologist, psychologists, and primary care researchers), share an interest in strategies to facilitate early treatment of inflammatory arthritis. The Consolidated Criteria for Reporting Qualitative Research (COREQ) guidelines [29] have been used in the reporting of the method and results of this study. Ethical approval was obtained from the University of Birmingham ethics committee (ethics code: ERN_16-0041).

### Involvement of patient research partners

The research team also involved six patient research partners (PRPs; with and without a diagnosis of RA) from the start of the project to ensure the project was relevant to patients with RA and to develop the study objectives. Preliminary results of the thematic analysis were also discussed with PRPs to aid data interpretation and discussion.

### Participants and study setting

Community pharmacies in the West Midlands were identified for participation in the study using the National Health Service (NHS) Choices ‘services near you’ website and approached by an initial visit or phone call from a researcher (GS) who introduced the study and provided the written invitation to take part and information about the study. In addition, pharmacies from the Jhoots pharmacy chain were approached through their links with the University of Birmingham School of Pharmacy and sent the study information via email. A convenience sampling technique [30] was used to select participants. This allowed the researchers to approach pharmacy staff through existing contacts and by visiting pharmacies to invite staff directly. Pharmacy staff interested in taking part were asked to contact the researcher conducting the interviews to arrange a suitable time and place for the interview. Participants did not receive an incentive for participation.

The inclusion criteria for participation were at least 18 years of age, working in a community pharmacy with regular interactions with members of the public, and ability to consent to and participate in an interview in the English language. Eligible participants thus included community pharmacists, pharmacy technicians, pharmacy assistants, pre-registration pharmacists in their training year and medicines counter assistants.

### Data collection

The interviews were conducted by GS, a female psychologist (PhD) with training and expertise in qualitative methodology. Data were collected through qualitative semi-structured interviews either face-to-face in the community pharmacy or over the phone and only GS and the participant were present during the interviews. Interviews were audio recorded and lasted between 30 and 45 min. No field notes were made by the interviewer.

Participants were made aware that the interviewer was a research fellow at the University of Birmingham and that she had a psychology rather than a pharmacy or medical background. The interviewer used an interview schedule which was informed by previous research [17, 31], expert clinical input and discussions with patient research partners (both patients with RA and members of the general public). Interview schedule questions were used as initial prompts for discussion, allowing interviewees to talk about their own experiences. The interview guide covered three main topics for discussion: (1) individuals visiting the pharmacy with joint symptoms and the advice the interviewee would give; (2) knowledge and perceptions of arthritis in general and RA in particular; and (3) advice and the role of pharmacy staff in encouraging appropriate help-seeking by patients with RA symptoms and other joint problems. The guide also includes a short vignette allowing interviewees to describe what actions they would take in the context of a patient presenting with RA symptoms (Table 1). Interviews were conducted over a 12-month period (2017–2018). Data collection continued until data saturation was achieved [32]. No repeat interviews were carried out.

**Table 1:** Interview guideline

General symptoms	How often do you think you are approached in the pharmacy by someone with joint problems/ someone asking for rubs or pain relief to deal with joint problems? What kind of advice to you generally give them? Do you ask questions about the symptoms, and if so, what do they involve? If someone came in with pain in their hands over the knuckle joints and in the small joints of the fingers, what would you think might be going on? What would you advise? Are there any questions you would ask?
Arthritis and Rheumatoid Arthritis	Tell me what you know about arthritis in general? Tell me what you know about Rheumatoid Arthritis (RA)? What symptoms would you associate with RA? What symptoms would you associate with osteoarthritis (OA)? What would be the main difference between RA and other joint problems? Is RA a serious condition? With whom do you associate arthritis/RA (who is the typical patient)? What would be the consequences of having RA for the day-to-day living of a patient? Do you think there are treatments available that would effetely treat RA? What kind of treatments do you know? Is early treatment important and if so with what and why?
Advice	What, according to you, is the role of the pharmacist in prevention/ early intervention of illnesses such as RA? Do you feel you know enough about RA to inform the public what to do if they experience possible symptoms? Would you like to receive more training (and if so, in what format)?

### Data analysis

The interviews were audio recorded, pseudo-anonymised and transcribed verbatim by a professional transcription company. The company transcribed all but one audio file. Due to the poor audio quality, that single recording was transcribed by the interviewer and two research assistants. The accuracy of the transcripts was checked by the interviewer (GS) through comparison with the original audio. Transcripts were not returned to the participants.

The transcripts were read in depth by two independent coders (who received training in the coding and analysis of interview transcripts) to familiarize themselves with the content and subsequently each coder initially coded the same two transcripts. Any disagreements were discussed with a third researcher (GS) and the coding adjusted after which each researcher coded half of the remaining transcripts. Coding was facilitated by NVivo 12 software [33] using the coding feature.

Coding was followed by thematic analysis [34]. Thematic analysis is a method for systematically identifying, organising, and deducting patterns of meaning (i.e., themes) across a dataset (e.g. transcripts from interviews). The researchers took an inductive approach towards the thematic analysis, allowing the data to determine the themes [34]. The codes were developed into initial themes using the codebook developed in NVivo with independent input from a further researcher (NI) and then discussed amongst the core research team (NI, GS, KR, and MF). The themes were revised after discussion and the team agreed on the final themes. Coded material was organised under the themes and subthemes using NVivo coding. Findings were presented using quotations to illustrate each theme identified. Participant numbers and pharmacy role were used to identify each quote. Participants did not provide feedback on the findings.

## Results

### Participant characteristics

Nineteen participants were recruited from 18 different pharmacies (17 community pharmacies and 1 pharmacy service within a GP practice). Participants were aged between 22 and 56 years (mean 32 years) with an almost equal male: female split (10:9). The participating staff included eight qualified pharmacists, (including 5 pharmacy managers), one pharmacy technician, three pharmacy assistants, and seven pre-registration pharmacists in either the second or third quarter of their pre-qualification training year (what is now called the foundation training year). Further participant characteristics are provided in Table 2. Three interviews were conducted in person and 16 by telephone. It is not possible to determine how many staff did not wish to participate as data regarding the numbers of staff the invitation was circulated to was not captured at all pharmacies. From the in-person visits it became clear that pharmacy staff declining the interview outright (at approximately 15 of the pharmacies visited) tended to be the pharmacist, with time frequently cited as a reason for non-participation.

**Table 2:** Participants’ characteristics

Participant number	Age range (years)	Gender	Role within the pharmacy	Range number of years working in pharmacy
1	Unknown*	Male	Pharmacist	11–15 years
2	50–60	Female	Pharmacy technician	16–20 years
3	40–50	Female	Pharmacist	Not known
4	30–40	Female	Pharmacist	6–10 years
5	50–60	Female	Pharmacist	21 + years
6	20–30	Female	Pre-registration	2nd quarter training year
7	40–50	Female	Pharmacist	21 + years
8	40–50	Male	Pharmacist	Not known
9	30–40	Male	Pharmacist	1–5 years
10	20–30	Male	Pharmacist	1–5 years
11	20–30	Female	Pharmacy assistant	1–5 years
12	30–40	Male	Pharmacy assistant	6–10 years
13	20–30	Female	Pharmacy assistant	1–5 years
14	20–30	Male	Pre-registration	2nd quarter training year
15	20–30	Male	Pre-registration	2nd quarter training year
16	20–30	Female	Pre-registration	2nd quarter training year
17	30–40	Male	Pre-registration	3rd quarter training year
18	20–30	Male	Pre-registration	3rd quarter training year
19	20–30	Male	Pre-registration	3rd quarter training year

*No age provided during interview

### Themes

Four main themes were identified: (1) Variations in perceptions and knowledge about RA. (2) The role of the pharmacy in increasing public awareness about RA. (3) The role of the pharmacy staff in facilitating access to the GP. (4) Practical considerations for pharmacy-based interventions.

#### Variations in perceptions and knowledge about RA

This theme covers pharmacy staff’s perceptions and knowledge of RA, its symptoms, and possible treatments. Several interviewees (both pharmacists and pre-registration pharmacists) gave an accurate description of RA as a progressive inflammatory disease (Table 3, Quote 1, further referred to as T3Q1), mentioned the fact that RA is an autoimmune disease (T3Q2) and accurately identified some of the main symptoms associated with it including joint pain and swelling:

Obviously, if their joints are swollen or quite obviously red and inflamed; even symmetrical swellings maybe that indicate rheumatoid arthritis of the joints, if there are any deformities. Any other symptoms that flag along with it, like feeling unwell, fever, etcetera. (P04, pharmacist).

**Table 3:** Quotes related to theme 1: variations in perceptions and knowledge about RA

Quote no	Quotes
1	*“It’s a progressive, degenerative disorder that we know will become worse if you don’t look at halting the disease progression; hence, the need for disease modifying anti-rheumatic drugs.”* P03*, Pharmacist
2	*“So rheumatoid arthritis, it's an auto-immune disease so the main symptoms we're looking at are joint pain, swelling, redness, warmth, maybe some fatigue, and fever.”* P16, Pre-registration pharmacist
3	*“I think the typical patient is probably over 50 but it can happen to a child. A child can have it and the parents are not aware. It doesn’t get diagnosed for a long time, I don’t think, in children because it’s not something you associate with children…”* P02, Pharmacy technician
*4*	*“The rheumatoid, is that autoimmune, is that right? So you want, more so get pain and swelling of the joints […] more so than osteoarthritis where that’s more wear and tear over years.”* P09, Pharmacist
5	*Hold on, osteoarthritis, that’s just the general term and rheumatoid arthritis is the more specific term that talks about the joints, mainly the fingers and everything. It’s an interchangeable term as far as I know”.* P19, Pre-registration pharmacist
6	*“I don’t think it’s specifically between men or women either, from my knowledge.”* P04, Pharmacist
7	*“100% yes of course, I think arthritis is definitely a serious condition, it’s affecting your day-to-day life, you’re in pain, you have to constantly rely on painkillers to go about your day.”* P14, Pre-registration pharmacist
8	*“Generally, in all honesty, if they come to me for RA, they’ve probably suffered for months, so another week or two is neither here nor there really, is it? I’m aware of the pressure on GP practice appointments.”* P01, Pharmacist
9	*“Yeah, I’d say the earlier you’ve started treating it the, it’s good ‘cause you can slow down like the progression.”* P10, Pharmacist
10	*“With rheumatoid arthritis, if the inflammation gets to a peak then the medication won’t have as much of an effect. Also, because it is physically debilitating, we always aim for prevention. So, in that way, I would say early treatment is preferred.”* P19, Pre-registration pharmacist
11	*“I don’t know whether other people are probably in the same boat as me but they probably weren’t as knowledgeable on rheumatoid arthritis and how important it is to intervene early.”* P04, Pharmacist
12	*“You’ve got things like Methotrexate, which seems to be the main thing. They also use NSAIDs and things like Sulfasalazine. Methotrexate would be the main one that springs to mind for rheumatoid arthritis. But they have to have blood level checks and toxicity checks for that.”* P05, Pharmacist
13	*“In terms of medication for that in particular, so we offer NSAIDs. Maybe a corticosteroid if it's an acute – maybe if we're injecting into the joint or DMARDs, so anything like methotrexate, Leflunomide, things like that. Or any biologic treatment after that, so Etanercept, Abatacept, that kind of stuff.”* P16, Pre-registration pharmacist

*P03 refers to participant 3 as described in Table 2. Idem for the other participant numbers

Some interviewees were further able to accurately describe the wide age range of patients affected with inflammatory arthritis (T3Q3, pharmacy technician). 

However, a number of pharmacists indicated not knowing much about RA or having gaps in their knowledge:

I mean I suppose I know a little bit about it […]. I mean I don’t know much about it other than more so the drug treatments that are available for it… (P09, pharmacist).

And some other pharmacy staff held misconceptions about RA and the causes of RA symptoms:

I think it's to do with the uric acid and stuff like that, when it's higher you get these joint pains and stuff. (P12, pharmacy assistant).

Moreover, whereas some interviewees could distinguish between RA and other MSK conditions such as osteoarthritis (OA) (T3Q4, pharmacist), others could not (T3Q5, pre-registration pharmacist). Further, not all interviewees were aware of the increased incidence rate of RA in women compared to men (T3Q6, pharmacist).

While some interviewees perceived RA as a serious condition associated with a reduced quality of life and an impact on physical functioning (T3Q7, pre-registration pharmacist), others did not perceive RA to be serious or in need of urgent attention, stating that waiting a few more weeks before seeking medical attention for these symptoms would not be problematic (T3Q8, pharmacist). Whereas some interviewees readily recognised the need for early DMARD treatment (T3Q9, pharmacist; T3Q10, pre-registration pharmacist), others did not (T3Q11, pharmacist). In general, pharmacists and pre-registration pharmacists were able to accurately describe the treatment of RA once it was diagnosed including DMARDs and non-steroidal anti-inflammatory drugs (NSAIDs) (T3Q12-Q13).

#### The role of the pharmacy in increasing public awareness about RA

Various interviewees shared their thoughts on the causes of patient delay in help-seeking and described a lack of public awareness of RA.

I suspect for the public perception there isn’t a whole lot. And when you say arthritis, they’re immediately thinking that they’re not old. I don’t think that the general public know much about rheumatoid arthritis. (P05, pharmacist).

Interviewees also recognised that this lack of knowledge about RA, the seriousness of RA and misperceptions around it being a disease of the elderly (e.g., Table 4; T4Q1, pharmacist) might be why symptomatic patients delayed seeking help.

**Table 4:** Quotes related to theme 2: the role of the pharmacy in increasing public awareness about RA

Quote no	Quotes
1	*“I think that’s something we’ve definitely seen. I mean we’ve [yeah] actually had one patient that just said, ‘Yeah, we, it’s, yeah, I thought it was actually part of getting old’.”* P10, Pharmacist
2	*“When it is early enough, it’s not severe enough for them to take action. And that is a problem that we have. That … even if there is a serious condition, what really would motivate someone to take some action, so clearly it really should start affecting their life. They don’t do anything.”* P08, Pharmacist
3	*“They don’t want to bother their GP. They don’t think the symptoms, particularly in the early stages, are specific enough to make them go the GPs.”* P05, Pharmacist
4	*“Yeah, they can’t be bothered to make another appointment at the doctors, and they feel like they might be embarrassing themselves just going for a little bit of pain you know in the joints.”* P07, Pharmacist
5	*“A lot don’t want to bother the GPs if they’re only having paracetamol.”* P05, Pharmacist
6	*“I know it’s hard for people to get a doctor’s appointment, which is what they keep saying.”* P02, Pharmacy technician
7	“*I think we’ve still got some of our leaflets. Yeah, national osteoporosis society. Am I at risk at osteoporosis and fractures? I mean any sort of learning materials like that, we go through them first in-house before we, you know, put them on the shelves for the patients ‘cause obviously we needed to get sourced up with everything*.” P09, Pharmacist
8	“*And also these posters and leaflets you can post to the different pharmacies and also we can give some leaflets and posters to, we can stick the posters inside the pharmacy and the people they can read and they can say and also they can have leaflets where they read information.”* P11*,* Pharmacy assistant

Some of the interviewed pharmacists indicated that patients might believe their condition was not serious enough to see a GP (T4Q2-Q3) and would only seek help if symptoms were so severe that they interfered with daily activities, thus delaying seeking help from their GP with early symptoms. Other interviewees thought patients may be embarrassed about seeking help for ‘*a little bit of pain*’ (T4Q4-Q5, pharmacists) or that they may find it difficult to get a GP appointment (T4Q6, pharmacy technician).

Several interviewees indicated ways in which they could be instrumental in increasing public awareness and informing their clients about RA.:

So it’s just a case of making sure that the conversations are happening so you don’t want to miss things so often it’s the case that the patient is seeking advice for the first time and if it is something like rheumatoid or something it is spotted and you are referring the right patients so I think we now have a bigger role in terms of awareness of arthritis and things, I'm not too sure how aware people are so… (P06, Pre-registration pharmacist).

Interviewees referred to leaflets and posters they have received for other diseases and suggested that equivalent materials would be useful for both increasing their own and their clients’ awareness of RA. (T4Q7, pharmacist; T4Q8, pharmacy assistant).

#### The role of pharmacy staff in facilitating access to the GP

This theme focused on the potential roles of community pharmacy staff in making positive interventions to signpost new onset RA patients towards GP consultations. Some of the interviewed pharmacists and pre-registration pharmacists explained how they would identify a potential new onset RA patient by assessing symptoms and asking about family history, referring patients towards their GPs if RA was suspected (Table 5; T5Q1–Q3). Some interviewees discussed how the role of pharmacists has changed with the focus being increasingly placed on clinical interactions with patients and not *“just dispensing the medication”* (T5Q4, pre-registration pharmacist), others described a lack of public awareness of pharmacists’ clinical knowledge and that they are underutilized:

I think it's about public perception as well because obviously I think they forget that we're also clinical pharmacists and we're trying to obviously change that and we—I think the public don't realise how much obviously we learn, and we know so we are an important port of call for things like that. (P16, pre-registration pharmacist).

**Table 5:** Quotes related to theme 3: the role of the pharmacy in facilitating access to the GP

Quote no	Quotes
1	*“If I did suspect rheumatoid arthritis in a patient, although I would offer to treat symptoms there, I would recommend that they saw a doctor”* P01, Pharmacist
2	*“Yes, I'd ask if they'd been diagnosed with—do they have any other medical conditions, things like that. So maybe if they have a history of it in the family.”* P16, Pre-registration pharmacist
3	*“Initially it’s just ruling out if the stiffness is worse in the mornings, those sorts of things.”* P06, Pre-registration pharmacist
4	*“I think it’s changed a lot recently because it’s kind of moved from us staying in the back just dispensing the medication, checking, to actually being out the front and making sure that we’re picking up things so it’s just a case of making sure that the conversations are happening so you don’t want to miss things so often it’s the case that the patient is seeking advice for the first time and if it is something like rheumatoid or something it is spotted and you are referring the right patients so I think we now have a bigger role in terms of awareness of arthritis.”* P06, Pre-registration pharmacist
5	*“I mean we usually like access the symptoms first and then obviously we refer it to the pharmacist and then like if he thinks that there’s anything more serious then he would always refer it to their GP.”* P13, Pharmacy assistant
6	*“In terms of stiffness in their hands or joint pain anywhere else in the body, at that point obviously I would […] just to keep an eye on generally what the patient is buying over the counter and spotting signs and clues as to there may be an underlying condition here.”* P15, Pre-registration pharmacist
7	*“So regarding joint pain the conversation I might have is more when I’m handing out pain medication and I strike up a conversation with the patient as to whether they’ve had the medication before and then it might go further into a conversation regarding their pain management.”* P07, Pharmacist
8	*“Sometimes people come and they ask for more medication even though the doctors prescribe something and then they ask for more tablets or more for example they just come for Co-codamol so many times even though they take medication at home they want some more tablets.”* P11, Pharmacy assistant

Furthermore, interviewed pharmacists described the role they thought other members of the pharmacy team (pharmacy assistants, technicians, and counter assistants) have to play as they are often the first to speak to patients.

I would say personally you don’t get referred that often because the actual counter assistants have the training now and so they’re like a filter. If somebody does come in to the pharmacy they’ll speak to those people first and if they can’t answer the questions, they’ll actually refer it to the pharmacist. (P07, pharmacist).

They indicated that when these staff members are unsure about the cause of the symptoms a patient is presenting with, they will generally refer the patient to a pharmacist who can further assess the patient and who in turn might refer the patient to the GP or advise the patient to seek help from a GP. This view was shared by other members of staff (e.g., T5Q5, pharmacy assistant).

Joint pain is the most common symptom of early RA and patients may seek advice about pain management from pharmacy staff. Indeed, interviewees indicated that this is when they are most likely to see a patient with inflammatory arthritis (T5Q6, pre-registration pharmacist).

Some interviewees described the considerations they would make when offering medications for the management of joint symptoms (T5Q7, pharmacist). Others explained that OTC medications should only be used temporarily to relieve symptoms while patients await their GP consultation:

Yes, in terms of giving medication OTC, that's just a temporary base, so what we can do, we can talk to the patient and get all the information and if we think this patient needs more than what we can do, we can refer to the GP. (P12, pharmacy assistant).

Interviewees further highlighted how they could flag patients who asked repeatedly for OTC medication for MSK symptoms including those of suspected RA and refer them onto the GP (T5Q08, pharmacy assistant).

#### Practical considerations for pharmacy-based interventions

This theme focused on the potential need for training of pharmacy staff and any other considerations needed to enhance opportunities for pharmacy staff to identify and signpost patients with new onset RA. Whereas some pharmacists had undertaken training on (rheumatoid) arthritis recently as part of their CPD, and felt they had a good level of knowledge about RA (Table 6; T6Q1), other interviewees indicated that although they had recently graduated and their current knowledge about RA was good, they were aware they would need more training in the future (T6Q2, pre-registration pharmacist). Some interviewees highlighted that there is currently no specific training requirement in relation to RA or other MSK conditions:

Yes, but only nine CPD’s a year. So, it might not even be musculoskeletal, it might be something different. So, it’s not something that says we have to learn musculoskeletal, it’s down to the pharmacist, so they might neglect it. (P17, pre-registration pharmacist).

**Table 6:** Quotes related to theme 4: Practical considerations for pharmacy based interventions

Quote no	Quotes
1	*“We have to do CPD, professionals we have to do CPD don’t we and also I did a community pharmacy diploma about three or four years ago and they have a section in that that you cover on arthritis and on pain management.”* P07, Pharmacist
2	“*Yes, absolutely. I think really for us the reason that I probably feel more confident and know a bit more is just because I've come out of university and we studied it. But I couldn't say confidently that five years down the line I'd know as much as I did now*.” P16, Pre-registration pharmacist
3	*“Yeah, obviously, I love training but I think it’s tricky … I’ve got three kids and I work 43 h a week. It’s tricky to fit it in.”* P01, pharmacist
4	*“I mean the dispensers or the healthcare counter staff are not really trained or aware of things that they should be picking up so they do refer to us”,* P06, Pre-registration pharmacist
5	*“If healthcare counter staff are just sending them away with pain relief and then they leave it there's always that downfall that they're not getting treated”,* P06, Pre-registration pharmacist
6	*“I think it's really good because it's like with the inhaler technique with us, once that came out about 10% of patients actually knowing how to use their inhaler and then pharmacists didn’t really know so then where is the knowledge coming from when they're prescribed it? So that was a big thing, I think things like this need to happen”* P06, Pre-registration pharmacist
7	*“A lot of other people would have the same situation where they can’t always attend. An e-learning course, promoted by CPPE, would be my preference, or something like that, because they promote emails and everybody uses those on a daily basis. That would be something to highlight this e-learning package. It’s got to be simple and it’s not very long.”* P04, Pharmacist
8	*“I would think a more face-to-face kind of approach. It’s a lot more useful. I mean you would need to do some preliminary work first and in the face-to-face interaction you might not cover all the ground at that stage but I think face-to-face is needed to consolidate it.”* P19, Pre-registration pharmacist
9	*“CCGs have a good, important role as well and so they could roll that out across the area; whether it’s in GP practices or community pharmacies. I’m sure they could. I don’t know whether they can promote that.”* P04, Pharmacist
10	*“Yes, we see people on a regular basis. We can see deterioration in some people and that goes for lots of other medical issues going on as well.”* P05, Pharmacist
11	*“We’re a village pharmacy, I’ve been there 25 years and so have half my staff, so we know the individual people that walk in, so we might initiate a conversation with somebody… You’ve initiated the conversation and given them the opportunity to speak to somebody about something that is occurring with them.”* P05, Pharmacist
12	*“If you're a locum and you're only in there for a day it’s very difficult to even get an idea of how often this patient comes in so it can be missed”* P15, Pre-registration pharmacist
13	*“I tend to do formal referrals when I think it’s urgent and so it helps to ease people through the system because sometimes, a letter from a pharmacist actually does carry a little bit of clout that would get you past Reception. When I’ve felt that someone needs to be seen quite urgently, I have given people referral letters to take to the Walk-In Centre.”* P01, Pharmacist
14	*“Sometimes if it’s like a medication review, so like an MUR … that we’ve done, we can do a referral letter … or normally it’s just, ‘Oh, you just need to make an appointment […] with your GP so they can’, you know, we leave that with the patient to do themselves […] arrange themselves. Or if we find it an emergency or often not an emergency, if it’s something that we can do for them that they feel involved, ‘Actually I know your surgery quite well or I know the girls at XXX and that’s our local doctor’s surgery, we can just pass them a message or we can just give them a call’, especially the times if it’s elderly patients or housebound patients maybe that have popped in on the off chance. I will give them a call, say, ‘You know, a doctor probably wants to […] his patient’, we’ve done that in the past as well.”* P09, Pharmacist

Whereas others stated that it was hard to find the time to do extra training (T6Q3, pharmacist).

Some of the pre-registration pharmacists stated that interventions made by pharmacy staff may vary depending on their clinical skills, and that technicians or counter staff did not have the clinical training to identify potential RA patients. They highlighted the importance of training for these members of the pharmacy team in addition to training provided for pharmacists, to ensure they can identify patients who might require requiring further referral rather than simply OTC medication (T6Q4-5). The lack of training of pharmacists themselves in clinical examination was also highlighted: *“It’s very varied because pharmacists have very little training in actual clinical examination”* (P01, pharmacist).

Some interviewees gave examples of training they had received in other disease areas, such as the inhaler technique service, which had led to improved patient outcomes (T6Q6, pre-registration pharmacist). Both pharmacists and pre-registration pharmacists further described their preferred training format which for some would be interactive online learning platforms (T6Q7, pharmacist) although others preferred face-to-face teaching (T6Q8, pre-registration pharmacist). Interviewees further suggested how training could be advertised to pharmacy staff to ensure training was delivered effectively to all community pharmacies (T6Q9, pharmacist).

Both pharmacists and pre-registration pharmacists identified that the setting of a pharmacy may influence the likelihood that patients with new onset RA would be identified. Smaller local pharmacies may see people on a regular basis and therefore pharmacy staff may initiate conversations to recognise new symptoms (T6Q10-12).

There was some discussion about the practicalities of how pharmacy staff might refer an individual they suspected of having RA to see their GP. Many would verbally advise their client to consult their GP:

It’s usually a verbal recommendation that I give and I go back to notes and if the patient is one of our patients then I would go to their notes and write down that I’ve had interaction with the patient, I told them to go and see the doctor, they were feeling this, this, this, I’ll make a note of it so that it’s in my record as well and kind of like for all purposes as well. But more importantly to know that I have referred them, and I know what’s happening now. (P14, pre-registration pharmacist).

However**,** some pharmacists described using a referral form when they believed the patient’s symptoms to be serious enough to require an urgent medial opinion (T6Q13, Pharmacist) or instead approaching the GP surgery directly if, for example, dealing with a vulnerable patient they believe needs to be seen urgently (T6Q14, pharmacist).

## Discussion

This qualitative study of the perceptions and knowledge of pharmacists and other members of the pharmacy team regarding RA, and their potential roles in signposting patients with suspected RA to their GPs identifies four important themes. Theme 1, ‘Variations in perceptions and knowledge about RA’, covers the variability of pharmacists’ perceptions and knowledge of RA, its symptoms, and possible treatments as well as the knowledge and perceptions of pre-registration pharmacists and other members of the pharmacy team. Theme 2 ‘The role of the pharmacy in increasing public awareness about RA’ highlights pharmacy staff recognising the importance of public awareness of RA and discusses how pharmacy staff can increase this. Theme 3 ‘The role of the pharmacy staff in facilitating access to the GP’ focuses on the potential roles of pharmacists and other members of the pharmacy team in making positive interventions to signpost patients with RA symptoms towards GP consultations. Finally, theme 4 ‘Practical considerations for pharmacy-based interventions’ discusses the potential need for training of pharmacy staff and other means by which to enhance opportunities for pharmacists to identify and signpost new onset RA patients.

These themes highlight both existing and future opportunities and a desire for positive intervention to improve access to care for patients presenting to community pharmacy settings with RA symptoms and to increase public and pharmacy team awareness of RA and musculoskeletal symptoms.

The findings show that pharmacists and pre-registration pharmacists feel that with appropriate support they could have a role to play in the early identification of suspect RA, early symptom management and appropriate signposting towards prompt and definitive treatment initiation in addition to their (future) role in the management of established disease. They also see a clear role for other members of the pharmacy team in identifying suspected RA cases. This study further demonstrates the potential roles pharmacists and other members of the pharmacy team can have in raising public awareness of RA. This aligns with current policy in many countries, including the UK, to expand pharmacy services [35–38] and to facilitate integration within primary care [39, 40]. With the correct knowledge and training, pharmacists are well positioned to use their clinical skills to identify and refer patients with a wide variety of ailments, including suspected RA, to their GPs where appropriate. The establishment of primary care networks and integrated care systems provide an important opportunity for collaboration between pharmacists and GPs, and improved care pathways for a range of conditions [41]. The evidence presented in this paper underlines the need to address RA within such systems.

### Implications for service development

Community pharmacy staff see people with, or at risk of, chronic conditions and see them longitudinally once diagnosed. Staff in local community pharmacies often build relationships with individual patients and their families and are well placed to identify new symptoms and discuss these with patients. There have been successful pharmacy based interventions in a number of chronic diseases such as diabetes, and cardiovascular diseases including heart failure, with pharmacy staff both identifying new patients and managing existing disease [27]. There is no reason why such a service cannot be implemented successfully for suspected RA as well. Indeed, our interview study has identified several ways in which pharmacists and other members of the pharmacy team can play an important role in reducing delays in initial GP consultation in the context of new onset RA, facilitating diagnosis within the 3-month window of opportunity. These include pharmacy assistants and medical counter assistants gaining skills to identify which MSK complaints should be referred onto the pharmacist on duty, additional training of pharmacists to enhance appropriate signposting and referral of suspected RA patients by pharmacists to the GP as well as increasing public awareness of RA and other MSK conditions through pharmacy-based interventions.

There is evidence of public misperceptions around RA, the symptoms, and the seriousness of the condition [17]. These findings could inform materials to be used in pharmacies to increase public awareness. This could take the form of leaflets for distribution within pharmacies and online resources the pharmacy staff could direct clients to. There is strong evidence that high quality pharmacy services can enhance the management of chronic conditions and thus facilitate better health outcomes [42]. For example, a systematic review showed that pharmacy-based interventions can contribute to improved treatment adherence and better disease control (e.g., blood pressure control, cholesterol management, and asthma control) [43] and a recent Cochrane review highlighted the potential positive impact of health‐promotion interventions on the behaviour of pharmacy staff, patients’ health behaviour, intermediate clinical outcomes, and their quality of life [44].

The interview data highlighted issues to address in educational interventions to enhance the role of pharmacists in the management of musculoskeletal symptoms. The interviewed pharmacists have variable knowledge of RA, the symptoms of RA and the need for early treatment. For example, although most interviewed pharmacists were aware of approaches to the treatment of established RA not all pharmacists were aware of the ideal window of opportunity for treatment initiation; therefore, training should highlight the need for prompt referral and treatment for new onset RA patients. Interestingly, the knowledge around RA of the interviewed pre-registration pharmacists who were all at least 4 or 5 months into their pre-qualification training year varied considerably too. Addressing these knowledge gaps through both university education and CPD presents an opportunity to enable effective signposting by (future) pharmacists which may lead to reduced treatment delay.

Among some of the interviewed pharmacists and pre-registration pharmacists, there was further evidence of confusion around differences between common MSK conditions such as RA and OA. Further training should address this and highlight when a GP referral is necessary and where (initial) self-management may be appropriate when patients present with MSK symptoms. MSK complaints currently account for around a third of GP consultations in the UK [45]. An estimated 18.8 million people are affected by MSK conditions in the UK. By 2030, 40% of the working age population are likely to have long-term conditions, with the prevalence of MSK conditions in the workforce predicted to increase [46]. Appropriate signposting and support of self-management directed by the pharmacist will be increasingly important for an effective primary care service.

Many of the pharmacy graduates in second or third quarter of their pre-registration training year appeared to have accurate knowledge about RA and the management of RA (sometimes more so than qualified pharmacists). This might be due to a stronger emphasis on RA and other MSK conditions during their formal undergraduate education. However, as discussed, misperceptions also existed amongst both qualified pharmacists and the pre-registration pharmacists. The current study was not designed to identify associations between participants’ degree of experience and their knowledge and ability to identify RA, however this would be an interesting area for future research and would help identify appropriate training needs for different career stages.

As indicated by some of the interviewees, for signposting to be delivered effectively in the context of RA, training is required for all pharmacy staff (i.e., not only pharmacists). Other pharmacy staff need to know when to refer to the pharmacist rather than simply advising symptomatic management with OTC medications. To do this effectively they would need to ask patients questions about their clinical history, identifying features strongly indicative of inflammatory arthritis and then seeking pharmacist input. Provision of RA specific flow charts with questions to elicit relevant symptoms may be a useful tool to aid pharmacy staff in making correct assessments.

Interviewees further suggested that awareness of the clinical knowledge of pharmacists and the role pharmacy staff can play in signposting and managing patients should be highlighted to both the general public and the wider primary care community. Various other studies have also confirmed that the public awareness of pharmacy services other than medicines supply is low [47–49]. However, the 2021 education and training standards for pharmacists will likely change public perception over the next decade as the pharmacist role is transformed [50]. The change in training standards means that all pharmacists will be independent prescribers upon completion of their pre-registration training (now referred to as the Foundation training year), thus increasing access to medicines for patients. This will require increased clinical and diagnostic skills to be developed in both undergraduate and post-graduate training [50] and in the long term increase the general public’s awareness and confidence in the clinical knowledge of pharmacists.

Finally, despite some pharmacies having guidelines and forms for direct referral to GPs or other health services, others rely on giving verbal advice to their clients to see a GP or other healthcare professional. Clear referral guidelines within a structured and nationwide referral system would benefit the evolving community care workforce, to ensure patients self-presenting at pharmacies can be formally referred to NHS services. Similarly, the NHS Community Pharmacist Consultation Service (CPCS), implemented in 2019, allows patients who request a GP appointment to be seen by a community pharmacist the same day for consultation. This consultation is documented and sent digitally to the patient’s GP. This service is designed for patients who have already decided that they need to see someone about their symptoms and if accessed by patients, this referral service and two-way communication between the pharmacist and the GP may improve delays in diagnosis and initiation of treatment, especially for those struggling to access primary care GP services [40]. There is clear potential for pharmacists to make appropriate referrals of patients with suspected RA to other NHS services and in doing so decrease patient delay and/ or other sources of delay in getting RA patients started on treatment within the window of opportunity.

### Limitations

A limitation to this study is that only one geographical region of the UK was studied and the perceptions and knowledge of pharmacists in other parts of the country might be different. Furthermore, although it was practical for the current study, convenience sampling may lead to selection bias, as it may not provide a representative sample of all the different types of pharmacy staff. For example, we were able to attract a relatively large sample of pre-registration pharmacists and pharmacists, but were not able to interview other staff, such as medicines counter assistants, who interacted with patients for advice about medications under the supervision of the pharmacist. Detailed data on work experience of participants was not recorded in the present study, limiting our ability to identify the impact of participants’ experience on their perceived ability to contribute to the identification of RA.

## Conclusion

Pharmacy staff believed they could play a greater role in the identification and management of new onset RA, and in raising public awareness of musculoskeletal conditions. There was wide variation amongst pharmacists and other pharmacy staff in their understanding of RA and of the importance of early treatment. This study highlights clear opportunities for intervention to provide training and resources for community pharmacy teams to raise public awareness about inflammatory joint symptoms and facilitate enhanced counselling and signposting/referral of patients presenting with such symptoms. We suggest that an effective intervention of this kind could contribute to a reduction in treatment delay and improved clinical outcomes.

## Data Availability

The datasets generated during and/or analysed during the current study are not publicly available due to the transcripts containing information that could compromise the privacy of research participants but are available from the corresponding author on reasonable request.

## Abbreviations

COREQ: Consolidated Criteria for Reporting Qualitative Research
CPD: Continuing professional development
DMARDs: Disease modifying anti-rheumatic drugs
EULAR: European Alliance of Associations for Rheumatology
GP: General practitioner
MSK: Musculoskeletal
NHS: National Health Service
NICE: National institute for health and care excellence
NSAIDs: Non-steroidal anti-inflammatory drugs
OA: Osteoarthritis
OTC: Over the counter
PIS: Participant information sheet
RA: Rheumatoid arthritis

## References

[1] Abhishek A, Doherty M, Kuo C-F, Mallen CD, Zhang W, Grainge MJ (2017). Rheumatoid arthritis is getting less frequent-results of a nationwide population-based cohort study. Rheumatology (Oxford).

[2] van der Linden MPM, le Cessie S, Raza K, van der Woude D, Knevel R, Huizinga TWJ (2010). Long-term impact of delay in assessment of patients with early arthritis. Arthritis Rheum.

[3] National Audit Office. Services for People with Rheumatoid Arthritis - National Audit Office (NAO) Report 2009 [cited 2020 14/12]. Available from: https://www.nao.org.uk/report/services-for-people-with-rheumatoid-arthritis/.

[4] NICE. Rheumatoid arthritis in adults: management NICE guidelines. [webpage]. 2018 [updated 12/10/2020; cited 2020 22/12]. Available from: https://www.nice.org.uk/guidance/ng100/chapter/Recommendations.

[5] Combe B, Landewe R, Daien CI, Hua C, Aletaha D, Álvaro-Gracia JM (2017). 2016 update of the EULAR recommendations for the management of early arthritis. Ann Rheum Dis.

[6] Raza K, Buckley CE, Salmon M, Buckley CD (2006). Treating very early rheumatoid arthritis. Best Pract Res Clin Rheumatol.

[7] Finckh A, Liang MH, van Herckenrode CM, de Pablo P (2006). Long-term impact of early treatment on radiographic progression in rheumatoid arthritis: a meta-analysis. Arthritis Rheum.

[8] van Nies JAB, Tsonaka R, Gaujoux-Viala C, Fautrel B, van der Helm-van Mil AHM (2015). Evaluating relationships between symptom duration and persistence of rheumatoid arthritis: does a window of opportunity exist? Results on the Leiden Early Arthritis Clinic and ESPOIR cohorts. Ann Rheum Dis.

[9] Smolen JS, Landewé RBM, Bijlsma JWJ, Burmester GR, Dougados M, Kerschbaumer A (2020). EULAR recommendations for the management of rheumatoid arthritis with synthetic and biological disease-modifying antirheumatic drugs: 2019 update. Ann Rheum Dis.

[10] Kumar K, Daley E, Carruthers DM, Situnayake D, Gordon C, Grindulis K (2007). Delay in presentation to primary care physicians is the main reason why patients with rheumatoid arthritis are seen late by rheumatologists. Rheumatology (Oxford).

[11] Raza K, Stack R, Kumar K, Filer A, Detert J, Bastian H (2011). Delays in assessment of patients with rheumatoid arthritis: variations across Europe. Ann Rheum Dis.

[12] De Cock D, Meyfroidt S, Joly J, Van der Elst K, Westhovens R, Verschueren P (2014). A detailed analysis of treatment delay from the onset of symptoms in early rheumatoid arthritis patients. Scand J Rheumatol.

[13] Stack RJ, Nightingale P, Jinks C, Shaw K, Herron-Marx S, Horne R (2019). Delays between the onset of symptoms and first rheumatology consultation in patients with rheumatoid arthritis in the UK: an observational study. BMJ Open.

[14] Stack RJ, Simons G, Kumar K, Mallen CD, Raza K (2013). Patient delays in seeking help at the onset of rheumatoid arthritis: the problem, its causes and potential solutions. Aging Health.

[15] Villeneuve E, Nam JL, Bell MJ, Deighton CM, Felson DT, Hazes JM (2013). Republished: a systematic literature review of strategies promoting early referral and reducing delays in the diagnosis and management of inflammatory arthritis. Postgrad Med J.

[16] Simons G, Belcher J, Morton C, Kumar K, Falahee M, Mallen CD (2017). Symptom recognition and perceived urgency of help-seeking for rheumatoid arthritis and other diseases in the general public: a mixed method approach. Arthritis Care Res (Hoboken).

[17] Simons G, Mason A, Falahee M, Kumar K, Mallen CD, Raza K (2017). Qualitative exploration of illness perceptions of rheumatoid arthritis in the general public. Musculoskeletal Care.

[18] Townsend A, Backman CL, Adam P, Li LC (2013). A qualitative interview study: patient accounts of medication use in early rheumatoid arthritis from symptom onset to early postdiagnosis. BMJ Open.

[19] Hassell K, Rogers A, Noyce P (2000). Community pharmacy as a primary health and self-care resource: a framework for understanding pharmacy utilization. Health Soc Care Community.

[20] NHS England. How your pharmacy can help 2019 [cited 2021 28/06]. Available from: https://www.nhs.uk/nhs-services/prescriptions-and-pharmacies/pharmacies/how-your-pharmacy-can-help/.

[21] Agomo C, Udoh A, Kpokiri E, Osuku-Opio J. Community pharmacists' contribution to public health: assessing the global evidence base. . Clin Pharmacist. 2018;10(4).

[22] Thomson K, Hillier-Brown F, Walton N, Bilaj M, Bambra C, Todd A (2019). The effects of community pharmacy-delivered public health interventions on population health and health inequalities: a review of reviews. Prev Med.

[23] Hayhoe B, Cespedes JA, Foley K, Majeed A, Ruzangi J, Greenfield G (2019). Impact of integrating pharmacists into primary care teams on health systems indicators: a systematic review. Br J Gen Pract.

[24] Marra CA, Grubisic M, Cibere J, Grindrod KA, Woolcott JC, Gastonguay L (2014). Cost-utility analysis of a multidisciplinary strategy to manage osteoarthritis of the knee: economic evaluation of a cluster randomized controlled trial study. Arthritis Care Res.

[25] Marra CA, Cibere J, Grubisic M, Grindrod KA, Gastonguay L, Thomas JM (2012). Pharmacist-initiated intervention trial in osteoarthritis: a multidisciplinary intervention for knee osteoarthritis. Arthritis Care Res.

[26] Marra CA, Cibere J, Tsuyuki RT, Soon JA, Esdaile JM, Gastonguay L (2007). Improving osteoarthritis detection in the community: Pharmacist identification of new, diagnostically confirmed osteoarthritis. Arthritis Care Res.

[27] Al Hamarneh YN, Marra C, Gniadecki R, Keeling S, Morgan A, Tsuyuki R (2021). R_x_IALTA: evaluating the effect of a pharmacist-led intervention on CV risk in patients with chronic inflammatory diseases in a community pharmacy setting: a prospective pre–post intervention study. BMJ Open.

[28] Thomas DR (2006). A general inductive approach for analyzing qualitative evaluation data. Am J Eval.

[29] Tong A, Sainsbury P, Craig J (2007). Consolidated criteria for reporting qualitative research (COREQ): a 32-item checklist for interviews and focus groups. Int J Qual Health Care.

[30] Marshall MN (1996). Sampling for qualitative research. Fam Pract.

[31] Simons G, Mallen CD, Kumar K, Stack RJ, Raza K (2015). A qualitative investigation of the barriers to help-seeking among members of the public presented with symptoms of new-onset rheumatoid arthritis. J Rheumatol.

[32] Faulkner SL, Trotter SP. Data Saturation. The International Encyclopedia of Communication Research Methods. p. 1–2.

[33] NVivo qualitative data analysis Software, version 12. 12 ed: QSR International Pty Ltd; 2012.

[34] Braun V, Clarke V. Thematic analysis. In: Cooper H, Camic PM, Long DL, Panter AT, Rindskopf D, Sher KJ, editors. APA handbook of research methods in psychology, Vol 2 Research designs: Quantitative, qualitative, neuropsychological, and biological 2: American Psychological Association; 2012. p. 57–71.

[35] NHS England. Next steps on the NHS five year forward view. : NHS England; 2017 [cited 2020 06/12]. Available from: https://www.england.nhs.uk/wp-content/uploads/2017/03/NEXT-STEPS-ON-THE-NHS-FIVE-YEAR-FORWARD-VIEW.pdf

[36] Erni P, von Overbeck J, Reich O, Ruggli M (2016). netCare, a new collaborative primary health care service based in Swiss community pharmacies. Res Social Adm Pharm.

[37] Thornley T, Esquivel B, Wright DJ, Dop Hvd, Kirkdale CL, Youssef E. Implementation of a Pharmacogenomic Testing Service through Community Pharmacy in the Netherlands: Results from an Early Service Evaluation. Pharmacy. 2021;9(1):38. 10.3390/pharmacy9010038 PMC7930936 33673111

[38] Gastelurrutia MA, Faus MJ, Martínez-Martínez F (2020). Primary health care policy and vision for community pharmacy and pharmacists in Spain. Pharm Pract (Granada).

[39] Raiche T, Pammett R, Dattani S, Dolovich L, Hamilton K, Kennie-Kaulbach N (2020). Community pharmacists' evolving role in Canadian primary health care: a vision of harmonization in a patchwork system. Pharm Pract (Granada).

[40] NHS England. NHS Community Pharmacist Consultation Service (CPCS) – Integrating Pharmacy into Urgent Care. : NHS England; 2020 [cited 2020 22/12]. Available from: https://www.england.nhs.uk/primary-care/pharmacy/community-pharmacist-consultation-service/.

[41] Integration and innovation: working together to improve health and social care for all In: Care DoHaS, editor. Online2021.

[42] Picton C. Measuring and improving patients’ experience of care: Report of a summit for pharmacy teams online: The Royal Pharmaceutical Society; 2016.

[43] Milosavljevic A, Aspden T, Harrison J (2018). Community pharmacist-led interventions and their impact on patients’ medication adherence and other health outcomes: a systematic review. Int J Pharm Pract.

[44] Steed L, Sohanpal R, Todd A, Madurasinghe VW, Rivas C, Edwards EA, et al. Community pharmacy interventions for health promotion: effects on professional practice and health outcomes. Cochrane Database of Syst Rev. 2019(12). 10.1002/14651858.CD011207.pub2 PMC6896091 31808563

[45] Margham T (2011). Musculoskeletal disorders: time for joint action in primary care. Br J Gen Pract.

[46] Versus Arthritis. The State of Musculoskeletal health 2019; Arthritis and other musculoskeletal conditions in numbers. 2019 [cited 2020 06/12]. Available from: https://www.versusarthritis.org/media/14594/state-of-musculoskeletal-health-2019.pdf

[47] Krska J, Morecroft CW (2010). Views of the general public on the role of pharmacy in public health. J Pharm Health Serv Res.

[48] Sinopoulou V, Summerfield P, Rutter P (2017). A qualitative study on community pharmacists' decision-making process when making a diagnosis. J Eval Clin Pract.

[49] Kember J, Hodson K, James DH (2018). The public's perception of the role of community pharmacists in Wales. Int J Pharm Pract.

[50] General Pharmaceutical Council. Standards for pharmacy education 2022 [Available from: https://www.pharmacyregulation.org/education/education-standards.

